# Assessment of Mouse Germinal Vesicle Stage Oocyte Quality by Evaluating the Cumulus Layer, Zona Pellucida, and Perivitelline Space

**DOI:** 10.1371/journal.pone.0105812

**Published:** 2014-08-21

**Authors:** Hong-Xia Zhou, Yu-Zhen Ma, Ying-Lei Liu, Ying Chen, Cheng-Jie Zhou, Sha-Na Wu, Jiang-Peng Shen, Cheng-Guang Liang

**Affiliations:** 1 The Key Laboratory of National Education Ministry for Mammalian Reproductive Biology and Biotechnology, Inner Mongolia University, Hohhot, Inner Mongolia, People’s Republic of China; 2 Inner Mongolia People’s Hospital, Hohhot, Inner Mongolia, People’s Republic of China; Institute of Zoology, Chinese Academy of Sciences, China

## Abstract

To improve the outcome of assisted reproductive technology (ART) for patients with ovulation problems, it is necessary to retrieve and select germinal vesicle (GV) stage oocytes with high developmental potential. Oocytes with high developmental potential are characterized by their ability to undergo proper maturation, fertilization, and embryo development. In this study, we analyzed morphological traits of GV stage mouse oocytes, including cumulus cell layer thickness, zona pellucida thickness, and perivitelline space width. Then, we assessed the corresponding developmental potential of each of these oocytes and found that it varies across the range measured for each morphological trait. Furthermore, by manipulating these morphological traits *in* *vitro*, we were able to determine the influence of morphological variation on oocyte developmental potential. Manually altering the thickness of the cumulus layer showed strong effects on the fertilization and embryo development potentials of oocytes, whereas manipulation of zona pellucida thickness effected the oocyte maturation potential. Our results provide a systematic detailed method for selecting GV stage oocytes based on a morphological assessment approach that would benefit for several downstream ART applications.

## Introduction

Oocyte quality plays a key role in embryo development and also affects the health of offspring. In humans, assisted reproductive technology (ART) is widely used in the treatment of infertility [1]. Retrieving and selecting germinal vesicle (GV) stage oocytes with high developmental potential is necessary for patients with ovulation problems. The ART procedure involves retrieval of GV stage oocytes from the ovary, followed by *in* *vitro* maturation (IVM), and then either *in* *vitro* fertilization (IVF) or intracytoplasmic sperm injection (ICSI). By administering human gonadotropin, the ovaries produce multiple oocytes that can be retrieved for ART [2], [3]. To reduce the incidence of medical, perinatal, and neonatal complications that are induced by multiple-gestation pregnancies, a strategy of single embryo transfer is used for ART [4]. Thus, selection of GV stage oocytes that possess the full potential to undergo fertilization and proper embryo development is a key step for successful human ART.

Previous studies have been conducted to better predict oocyte quality based on molecular markers and cell morphology. Granulosa cells (GCs) and cumulus cells, which are closely attached to oocytes, have been widely used in oocyte quality prediction, and genes expressed specifically in GCs have been used as markers to predict the developmental potential of GV stage oocytes [5]. Transcriptome profiles of cumulus cells and GCs have also been systematically analyzed to better understand oocyte maturation, fertilization, and embryo quality, and to better predict pregnancy outcome [6], [7]. However, extraction of RNA or protein for such analyses necessitates the destruction of the oocyte, making this technique impractical in the clinic. Recently, genome analysis of the first polar body (PB1) and second polar body (PB2) extruded from a single human oocyte has been performed, thus providing a promising method for preimplantation genomic screening [8].

The quality of oocytes can also be analyzed using stereomicroscopy to observe their well-characterized morphological traits, such as the polar body (PB) extrusion, cumulus cell layer, zona pellucida (ZP), and perivitelline space. Cumulus cells play an essential role during oocyte maturation [9]. In ovarian follicles, cumulus cells and GCs communicate with oocytes to support proper oocyte growth [10], which is important for fertilization and embryonic development [11]. A previous study found that human cumulus cells do not affect oocyte nuclear maturation, but instead affect cytoplasmic maturation and subsequent embryonic development [12]. Another study showed that inhibition of cumulus cell apoptosis improves oocyte quality and the embryo development rate [13]. Thus, simply evaluating the cumulus layer surrounding the oocyte is an efficient and reliable prognostic tool that can be used in embryology laboratories [14].

As ovarian follicles grow, follicular fluid that is secreted by follicular GCs and cumulus cells increases, leading to the formation of the ZP [15]. The ZP plays a key role in preventing polyspermy during fertilization [16]. Abnormalities in the ZP can lead to failure of fertilization, implantation, and pregnancy [17], [18]. Mouse ZP is comprised of three types of glycoproteins: ZP1, ZP2, and ZP3 [19]. A recent study showed that oocytes lacking, or with incomplete synthesis of, ZP1, ZP2, or ZP3 cannot be fertilized [20], [21]. Furthermore, ZP thickness variation has also been shown to be a strong predictor of pregnancy rates in human IVF treatment [22].

The perivitelline space is a subcellular structure that exists between the ZP and the oocyte plasma membrane that is rich in extracellular matrix components, which are essential for fertilization, implantation, and embryo development. Reports regarding the correlation between the size of the perivitelline space and embryo quality have been controversial. Studies reported that there is a correlation between the size of the perivitelline space, the presence of granulation, and subsequent embryo quality [23], [24], but not with implantation rates and clinical pregnancy [23]. However, another study found that the presence of coarse granules is associated with lower pregnancy and implantation rates [25]. Another study found that increased perivitelline space correlates with low fertilization rates and compromised pronuclear morphology, but has no further effect on embryo quality [26]. Therefore, further investigation into the relationship between the morphological characteristics of the perivitelline space and oocyte quality is warranted to resolve these controversies.

Thorough morphological analyses of the cumulus cell layers, ZP, and perivitelline space of mature oocytes should provide feasible criteria to predict their developmental potential. In this study, we investigated the relationship of these morphological analyses of GV stage oocytes to their potential to undergo maturation, fertilization, and subsequent embryo development. We also manipulated the GV stage oocytes by manually removing cumulus layers, partially digesting the ZP, and widening the perivitelline space to better understand the functional implications of these morphological variations. Our study provides systematic, accurate criteria for selecting GV stage oocytes with enhanced developmental potential, which would improve several downstream ART applications.

## Materials and Methods

### Ethics Statement

All studies adhered to procedures consistent with the National Research Council Guide for the Care and Use of Laboratory Animals and were approved by the Institutional Animal Care and Use Committee at Inner Mongolia University. All mice treatments were performed with efforts to minimize suffering and were killed by cervical dislocation.

### Oocyte Collection, Maturation, Fertilization, Parthenogenetic Activation, and Embryo Culture

Female (B6D2) F1 mice at 4–8 weeks of age were purchased from Vital River Laboratories (Beijing, China) and used for sample collections. The mice were maintained in a temperature and light controlled, pathogen-free space (22°C, 14 hours light/10 hours dark, with lights on at 6∶00 AM). The animals had free access to food and water. The mice were randomly allocated to each experimental group. All chemicals and media were purchased from Sigma Aldrich Company (St. Louis, MO), unless stated otherwise. GV stage cumulus oocyte complexes (COCs) were collected 48 hours after injection of pregnant mare serum gonadotropin (PMSG, Sansheng, Ningbo, China) by puncturing the follicles of ovaries. For experiments using GV stage denuded oocytes (DOs), cumulus cells were removed from the oocytes by gentle pipetting in M2 medium supplemented with 0.3 mg/ml hyaluronidase [27].

The maturation of COCs was conducted in MEMα medium (Gibco, New York, NY) supplemented with 10 ng/ml EGF and 0.01 AU/ml FSH. The maturation of DOs was conducted in Chatot-Ziomet-Bavister (CZB) medium containing 5% FBS. Drops of 50 µl maturation medium containing 20 COCs or DOs were covered with paraffin oil and incubated under a humidified atmosphere of 5% CO_2_ at 37°C. The PB1 extrusion was observed at 14 hours after maturation. The matured oocytes were used for fertilization or activation.

The COCs matured *in* *vitro* were used for IVF. The spermatozoa were collected from the cauda epididymis of adult male (B6D2) F1 mice at 10–14 weeks of age. The sperm suspension was capacitated for 2 hours in 200 µl of T6 medium supplemented with 4 mg/ml BSA. The metaphase II (MII) oocytes were incubated with spermatozoa for 6 hours. The sperm concentration used for fertilization was 1×10^6^/ml.

In order to measure the thicknesses of the ZP and the widths of perivitelline space, cumulus layers attached to the oocytes need to be removed first. Oocytes without cumulus cells, *i.e.*, DOs, have much lower ratio of *in* *vitro* fertilization and subsequent embryo development. However, oocyte parthenogenetic activation is not interfered by the absence of cumulus. Thus, for experiments of checking ZP thickness or perivitelline space, parthenogenetic activation was used to evaluating oocyte quality. Parthenogenetic embryos were generated by incubating the DOs for 6 hours in calcium-free CZB-Glutamine medium supplemented with 10 mM SrCl_2_ and 5 µg/ml cytochalasin B [28], [29].

The zygotes generated from IVF or parthenogenetic activation were cultured in CZB medium under a humidified atmosphere of 5% CO_2_ at 37°C for the first two days, and then transferred to CZB medium supplemented with 5.5 mmol/L glucose for the remaining time in culture. The embryos were checked at 24, 48, 72, and 96 hours post insemination or parthenogenetic activation to record the 2-cell, 4-cell, morula, and blastocyst development, respectively.

### Dispelling Cumulus Cell layer

To dispel the cumulus layer of COCs, the cumulus cells were partially removed from the oocytes by gentle pipetting in M2 medium supplemented with 0.3 mg/ml hyaluronidase. The thickness of the cumulus layer was measured using an inverted microscope with a 100× objective and analyzed by CellSens Standard software (Olympus, Japan).

### ZP Digestion

To digest the ZP, DOs were incubated in acidic tyrode solution (pH = 2.4) and then monitored using a stereo microscope. The digestion was terminated by moving the oocytes to M2 medium, followed by two washes. The diameter of the ZP was measured using an inverted microscope with a 100× objective and analyzed by CellSens Standard software.

### Perivitelline Space Enlargement

The perivitelline space was enlarged by removing part of the ooplasm from oocytes. GV stage oocytes were incubated in M2 medium containing 5 µg/ml cytochalasin B (CB), 0.2 µg/ml demicolcine, and 0.1 mmol/l 3-Isobutyl-1-methylxanthine (IBMX). A pipet with a 7.5-µm inner diameter was mounted on a piezo instrument for the ooplasm extraction. The perivitelline space became larger upon partial removal of the ooplasm. To minimum the harm of ooplasm extraction to oocyte, we remove less ooplasm as far as possible to achieve the desired perivitelline space. The diameter of the perivitelline space was measured using an inverted microscope with a 100× objective and analyzed by CellSens Standard software. Following treatment, the oocytes were washed with maturation medium at least three times to remove CB, demicolcine, and IBMX.

### Experimental Design

The thicknesses of the cumulus cell layer (C) in GV stage COCs were classified into three categories: C≥50 µm, 30 µm≤C<50 µm, and C<30 µm. For experiments using COCs with dispelled cumulus layers, COCs with C≥50 µm were treated to make C<30 µm.

The thicknesses of the ZP (Z) in GV stage oocytes were classified into three categories: Z≥8 µm, 5 µm≤Z<8 µm, and Z<5 µm. For the ZP digestion experiments, oocytes with Z≥8 µm were treated to make 5 µm≤Z<8 µm.

The widths of the perivitelline space (P) in GV stage oocytes were classified into three categories: P≥5 µm, 1 µm≤P<5 µm, and P<1 µm. For the perivitelline space enlargement experiments, oocytes with P<1 µm were treated to make 1 µm≤P<5 µm.

### Statistical Analysis

Each group consisted of at least 50 oocytes, with a minimum of three replicates for each experiment. The ratio of oocytes with PB1 to total GV oocytes used for maturation was denoted as the oocyte maturation rate. The ratio of 2-cell embryos to matured oocytes used for fertilization or activation was denoted as the fertilization or activation rate. The ratio of 4-cell embryos, morula, or blastocyst to 2-cell embryos was denoted as the 4-cell, morula, or blastocyst rate, respectively. The statistical significance of differences in development was evaluated using a χ^2^ test with IBM SPSS Statics 19.0 software (http://www-01.ibm.com/software/analytics/spss/). A probability value of P<0.05 was considered statistically significant.

## Results

### Effect of cumulus layer thickness on oocyte maturation, fertilization, and early embryo development

**Figure 1:**
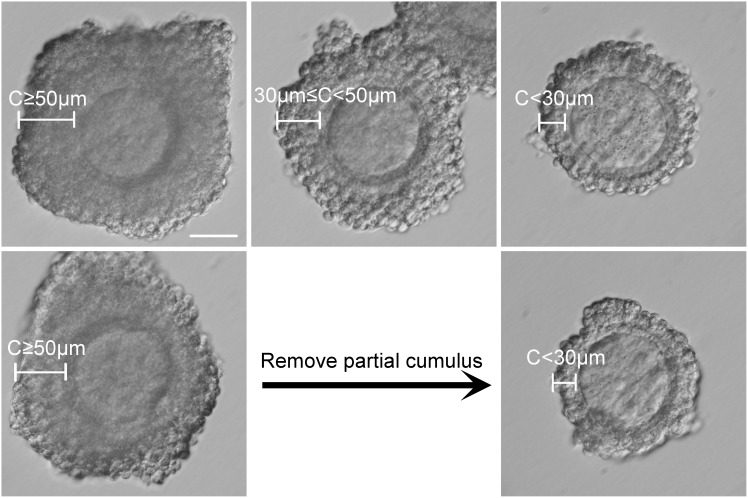
GV stage oocyte classification based on thickness of the cumulus layers. GV stage COCs were collected from ovaries from mice following administration of PMSG. The COCs were classified into three categories according to the thickness of their cumulus layers (C): COCs with C≥50 µm (upper line, left), COCs with 30 µm≤C<50 µm (upper line, middle), COCs with C<30 µm (upper line, right). For the experiment using COCs with dispelled cumulus layers, COCs with C≥50 µm (bottom line, left) were treated using gentle pipetting to generate C<30 µm (bottom line, right). Scale = 50 µm.

To evaluate the effect of cumulus layer thickness on oocyte developmental potential, we first divided the GV stage COCs into three categories according to the thicknesses of their surrounding cumulus layer (C). The COCs that were in the category of C≥50 µm, had more than three layers of cumulus cells, whereas the COCs that had two or three cumulus cell layers fell into the category of 30 µm≤C<50 µm, and the COCs with less than two layers of cumulus cells fell into the category of C<30 µm (Figure 1, upper line).

To determine whether the GV stage COCs with varying amounts of cumulus cell layers have the same capacity to undergo *in* *vitro* maturation, we evaluated the oocyte maturation rate of COCs from each category of cumulus layer thickness. The oocyte maturation rate was quantified as the ratio of the number of oocytes with PB1 to the number of GV stage oocytes used for maturation. We found that all of the COCs with C≥50 µm were able to progress to the metaphase II (MII) stage (100%, 111/111), thus showing the highest rate of oocyte nuclear maturation. The COCs with 30 µm≤C<50 µm showed a slightly decreased rate in comparison, with 94.8% (255/269) showing PB1 extrusion (P<0.05). The lowest oocyte nuclear maturation rate was observed in the COCs with C<30 µm, with only 85.3% (116/136) displaying PB1 extrusion (P<0.01) (Table 1). These data suggest that a thicker cumulus cell layer promotes *in* *vitro* oocyte maturation.

**Table 1:** Effect of cumulus layer thickness on oocyte maturation and early embryo development.

thickness ofcumuluslayers (C)	maturation	Repeattimes	PB1%(oocytes withPB1/Total GVoocytes formaturation)	2-cell%(2-cells/maturedoocytes forfertilization)	4-cell%(4-cells/2-cells)	morula%(morula/2-cells)	blastocyst%(blastocysts/2-cells)
C≥50 µm	*In vitro* COC	3	100.00 (111/111)^a^	71.88 (115/160)^a^	73.91 (85/115)^a^	73.91 (85/115)^a^	64.35 (74/115)^a^
30 µm≤C<50 µm	*In vitro* COC	3	94.80 (255/269)^b^	55.56 (105/189)^b^	40.95 (43/105)^b^	40.95 (43/105)^b^	26.67 (28/105)^b^
C<30 µm	*In vitro* COC	3	85.30 (116/136)^c^	22.22 (36/162)^c^	2.78 (1/36)^c^	2.78 (1/36)^c^	0 (0/36)^c^

Within a column, ratio without a common letter (a–c) differs (P<0.05).

To determine the relationship between cumulus layer thickness and fertilization, we quantified the fertilization rate of the COCs as the ratio of the number of 2-cell embryos obtained to the number of matured oocytes used for fertilization. The COCs with C≥50 µm displayed the highest fertilization rate (2-cell: 71.88% [115/160]), whereas COCs with 30 µm≤C<50 µm showed a decreased fertilization rate (2-cell: 55.56% [105/189]) (P<0.01). The COCs with C<30 µm showed the lowest fertilization rate, with only 22.22% (36/162) of the matured oocytes progressing to the 2-cell stage (P<0.01) (Table 1). These data suggest that a thicker cumulus layer not only promotes oocyte maturation, but also enhances fertilization.

Similar to the effects observed on maturation and fertilization, cumulus layer thickness of COCs also showed effects on *in* *vitro* embryo development rates, which was quantified as the ratio of the number of 4-cell, morula, or blastocyst stage embryos obtained to the number of 2-cell stage embryos. The COCs with C≥50 µm showed the highest rate of embryo development (4-cell: 73.91% [85/15], morula: 73.91% [85/115], blastocyst: 64.35% [74/115]). The COCs with 30 µm≤C<50 µm showed an intermediate rate of embryo development (4-cell: 40.95% [43/105], morula: 40.95% [43/105], blastocyst: 26.67% [28/105]), whereas the COCs with C<30 µm showed the lowest rate of embryo development. From this category, only 2.78% (1/36) of the fertilized zygotes developed to the 4-cell and morula stages, and no embryos progressed to the blastocyst stage (P<0.01) (Table 1). These data further suggest that increased cumulus cell layers are important for promoting the developmental potential of oocytes.

To determine whether the relationship of the cumulus layer thickness to the developmental potential of oocytes is causal, we manually removed cumulus cell layers from oocytes and investigated the effects on maturation, fertilization, and embryo development. More specifically, we removed cumulus cells from oocytes with C≥50 µm to reduce them to C<30 µm to test our hypothesis (Figure 1, bottom line). As shown in Table 2, following removal of cumulus cell layers by gentle pipetting, the rate of PB1 extrusion, or oocyte maturation, decreased from 98.39% (245/249) to 95.82% (229/239). However, this decrease was not statistically different, suggesting that cumulus layer removal does not affect oocyte maturation potential. Interestingly, partial removal of cumulus cell layers from COCs significantly decreased the rates of fertilization and embryo development compared with the control group, which contained at least three cumulus cell layers. The 2-cell stage rate, or fertilization rate, decreased from 68.69% (215/313) to 49.07% (159/324), the 4-cell from 70.70% (152/215) to 45.28% (72/159), the morula from 72.56% (156/215) to 44.03% (70/159), and the blastocyst from 61.40% (132/215) to 28.93% (46/159) (P<0.01) (Table 2). These results further demonstrate the importance of cumulus layer thickness to the developmental potential of oocytes.

**Table 2:** Effect of cumulus cell layer removal on oocyte maturation and early embryo development.

thickness ofcumuluslayers ©	maturation	Repeattimes	PB1%(oocytes withPB1/TotalGV oocytesfor maturation)	2-cell%(2-cells/maturedoocytes forfertilization)	4-cell%(4-cells/2-cells)	morula%(morula/2-cells)	blastocyst%(blastocysts/2-cells)
C≥50 µm	*In vitro* COC	3	98.39 (245/249)^a^	68.69 (215/313)^a^	70.70 (152/215)^a^	72.56 (156/215)^a^	61.40 (132/215)^a^
Remove partial cumulus to make C<30 µm	*In vitro* COC	3	95.82 (229/239)^a^	49.07 (159/324)^b^	45.28 (72/159)^b^	44.03 (70/159)^b^	28.93 (46/159)^b^

Within a column, ratio without a common letter (a–b) differs (P<0.01).

### Effect of ZP thickness on oocyte maturation, activation, and early embryo development

**Figure 2:**
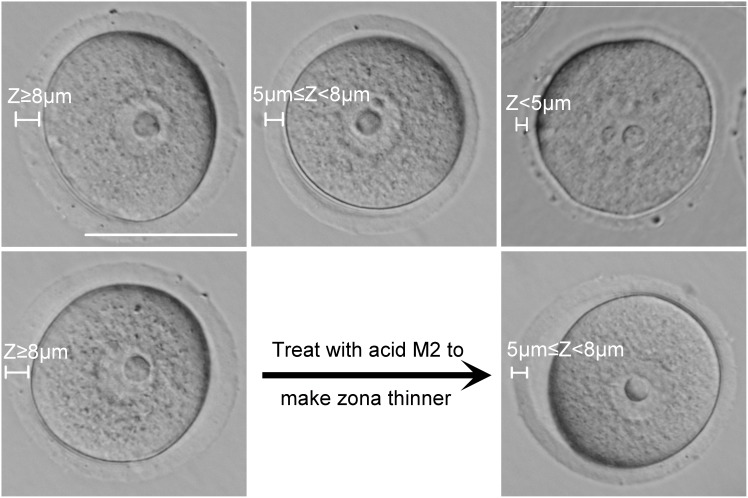
GV stage oocyte classification based on thickness of the ZP. GV stage COCs were collected from ovaries from mice following administration of PMSG. The COCs were classified into three categories according to the thickness of their ZP (Z): COCs with Z≥8 µm (upper line, left), COCs with 5 µm≤Z<8 µm (upper line, middle), COCs with Z<5 µm (upper line, right). For the ZP digestion experiment, oocytes with Z≥8 µm (bottom line, left) were treated with tyrode solution to generate 5 µm≤Z<8 µm (bottom line, right). Scale = 50 µm.

To determine if ZP thickness of GV stage oocytes affects maturation, activation, and embryo development, we classified oocytes into three categories according to their ZP thickness (Z): Z≥8 µm, 5 µm≤Z<8 µm, Z<5 µm. The typical morphology of oocytes in each group is shown in Figure 2 (upper line).

First, we determined the rate of PB1 extrusion, or ooctye maturation, for oocytes within each category of ZP thickness. As shown in Table 3, oocytes with Z≥8 µm showed a PB1 extrusion rate of 83.14% (217/261), whereas oocytes with 5 µm≤Z<8 µm showed a higher PB1 extrusion rate of 88.92% (337/379) (P<0.05). On the other hand, only 2.00% (1/50) of the oocytes with Z<5 µm matured, which is a much lower rate than those of the other ZP thickness categories (P<0.01) (Table 3), suggesting that ZP thickness affects oocyte maturation potential.

**Table 3:** Effect of ZP thickness on oocyte maturation and early embryo development.

thickness ofzonapellucida (Z)	maturation	Repeattimes	PB1%(oocytes withPB1/TotalGV oocytesfor maturation)	2-cell%(2-cells/maturedoocytes foractivation)	4-cell%(4-cells/2-cells)	morula%(morula/2-cells)	blastocyst%(blastocysts/2-cells)
Z≥8 µm	*In vitro* DO	3	83.14 (217/261)^a^	35.32 (71/201)^a^	15.49 (11/71)^a^	12.68 (9/71)^a^	0 (0/71)^a^
5 µm≤Z<8 µm	*In vitro* DO	3	88.92 (337/379)^b^	28.91 (85/294)^a^	16.47 (14/85)^a^	15.29 (13/85)^a^	4.71 (4/85)^a^
Z<5 µm	*In vitro* DO	3	2.00 (1/50)^c^	0 (0/1)^b^	N/A	N/A	N/A

Within a column, ratio without a common letter (a–c) differs (P<0.05).

Next, we evaluated the activation and embryo development rates for oocytes within each category of ZP thickness. There was no statistical difference in the activation rates between the oocytes with Z≥8 µm and 5 µm≤Z<8 µm (35.32% [71/201] versus 28.91% [85/294]). For the oocytes with Z<5 µm, only one oocyte matured, but failed to activate. Therefore, the activation rate of zero was significantly lower than that of oocytes with Z ≥5 µm (P<0.01). The subsequent embryo development rate did not show any statistical difference between oocytes with Z≥8 µm and 5 µm≤Z<8 µm (4-cell: 15.49% [11/17] versus 16.47% [14/85], morula: 12.68% [9/71] versus 15.29% [13/85], blastocyst: 0 [0/71] versus 4.71% [4/85]) (Table 3).

Because our data showed that oocytes with 5 µm≤Z<8 µm have a higher potential to mature than that of oocytes with Z≥8 µm, we hypothesized that reducing the ZP thickness of oocytes with Z≥8 would improve oocyte maturation. Oocytes with Z≥8 were incubated with acidic tyrode solution to partially digest the ZP and generate oocytes with 5 µm≤Z<8 µm (Figure 2, bottom line). Then, we evaluated the rates of oocyte maturation and activation, and the rates of 4-cell, morula, and blastocyst embryo development, and compared them between oocytes without acidic tyrode solution treatment. As shown in Table 4, following acidic tyrode solution treatment to digest the ZP, the rate of oocyte maturation increased (Z≥8 µm: 85.71% [108/126] versus 5 µm≤Z<8 µm: 93.68% [163/174]) (P<0.05). However, ZP digestion did not improve activation and embryo development rates (2-cell: 33.65% [35/104] versus 41.61% [67/161], 4-cell: 14.29% [5/35] versus 28.36% [19/67], morula: 11.43% [4/35] versus 20.90% [14/67], blastocyst: 0 [0/35] versus 2.99% [2/67]) (Table 4). These results are similar to the observations of the unmanipulated oocytes, and suggest that there may be an optimal ZP thickness for oocyte maturation (5 µm≤Z<8 µm).

**Table 4:** Effect of ZP digestion on oocyte maturation and early embryo development.

thickness ofzonapellucida (Z)	maturation	Repeattimes	PB1%(oocytes withPB1/TotalGV oocytesfor maturation)	2-cell%(2-cells/maturedoocytes foractivation)	4-cell%(4-cells/2-cells)	morula%(morula/2-cells)	blastocyst%(blastocysts/2-cells)
Z≥8 µm	*In vitro* DO	3	85.71 (108/126)^a^	33.65 (35/104)^a^	14.29 (5/35)^a^	11.43 (4/35)^a^	0 (0/35)^a^
Digest partial zona to make 5 µm≤Z<8 µm	*In vitro* DO	3	93.68 (163/174)^b^	41.61 (67/161)^a^	28.36 (19/67)^a^	20.90 (14/67)^a^	2.99 (2/67)^a^

Within a column, ratio without a common letter (a–b) differs.

### Effect of perivitelline space width on oocyte maturation, activation, and early embryo development

**Figure 3:**
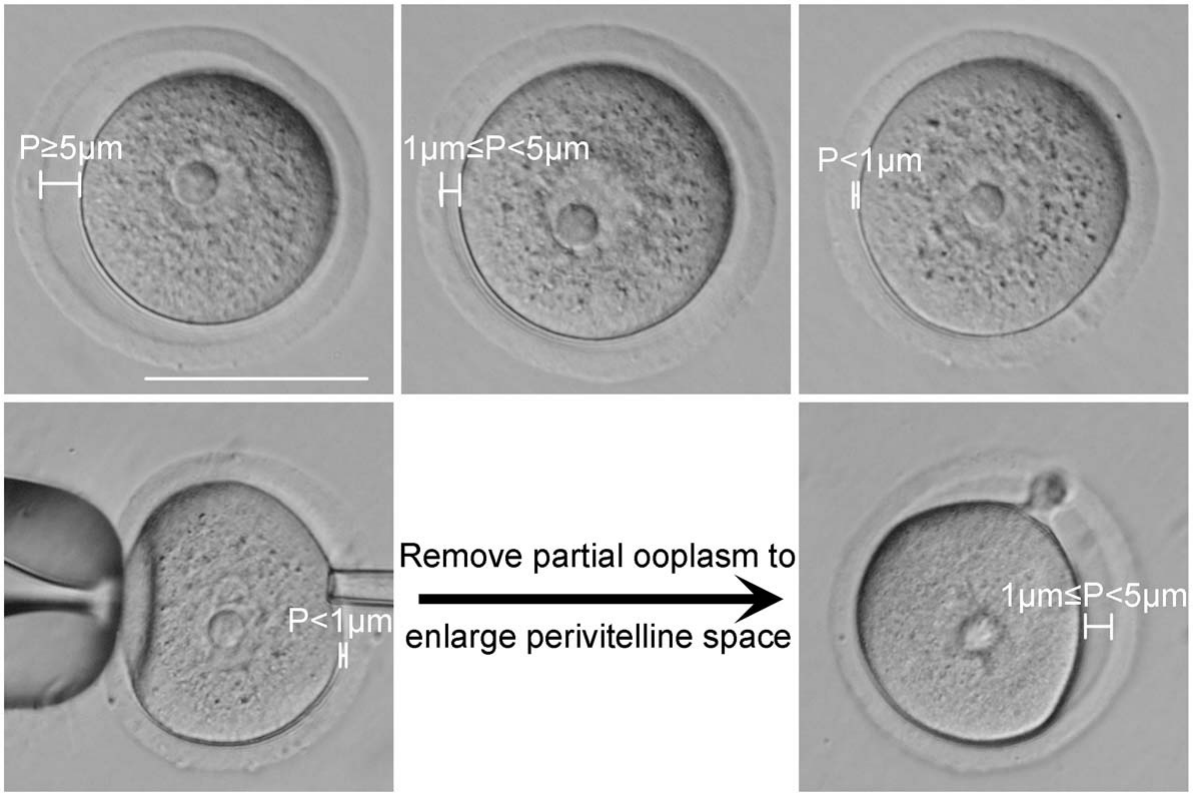
GV stage oocyte classification based on width of the perivitelline space. GV stage COCs were collected from ovaries from mice following administration of PMSG. The COCs were classified into three categories according to the width of their perivitelline space (P): COCs with P≥5 µm (upper line, left), COCs with 1 µm≤P<5 µm (upper line, middle), COCs with P<1 µm (upper line, right). For the perivitelline space enlargement experiment, oocytes with P<1 µm (bottom line, left) were treated by extracting ooplasm to generate 1 µm≤P<5 µm (bottom line, right). Scale = 50 µm.

To evaluate the effect of perivitelline space width on oocyte developmental potential, we first divided oocytes into three categories according to the width of their perivitelline space (P): P≥5 µm, 1 µm≤P<5 µm, P<1 µm. The typical morphology of oocytes in each group is shown in Figure 3 (upper line).

First, we determined the rate of PB1 extrusion, or oocyte maturation, within each category of perivitelline space width. As shown in Table 5, oocytes with P≥5 µm had a higher rate of PB1 extrusion than that of oocytes with 1 µm≤P<5 µm (92.61% [188/203] versus 72.89% [121/166]) (P<0.01). Oocytes with P<1 µm had a very low maturation rate, with only 1.92% (1/52) of the oocytes displaying PB1 extrusion, which is much lower than that of the other two categories (P<0.01). These data suggest that a wider perivitelline space supports oocyte maturation.

**Table 5:** Effect of perivitelline space width on oocyte maturation and early embryo development.

width ofperivitellinespace (P)	maturation	Repeattimes	PB1%(oocytes withPB1/TotalGV oocytesfor maturation)	2-cell%(2-cells/maturedoocytes foractivation)	4-cell%(4-cells/2-cells)	morula%(morula/2-cells)	blastocyst%(blastocysts/2-cells)
P≥5 µm	*In vitro* DO	4	92.61 (188/203)^a^	35.16 (64/182)^a^	7.81 (5/64)^a^	4.69 (3/64)^a^	3.13 (2/64)^a^
1 µm≤P<5 µm	*In vitro* DO	4	72.89 (121/166)^b^	51.02 (50/98)^b^	32.73 (18/55)^b^	16.36 (9/55)^b^	7.27 (4/55)^a^
P<1 µm	*In vitro* DO	4	1.92 (1/52) ^c^	0 (0/1) ^c^	N/A	N/A	N/A

Within a column, ratio without a common letter (a–c) differs (P<0.05).

Next, we assessed the rate of activation, and the rates of 4-cell, morula, and blastocyst embryo development according to each category. Interestingly, oocytes with 1 µm≤P<5 µm had a higher rate of activation (51.02% [50/98]) compared to that of oocytes with P≥5 µm (35.16% [64/182]) (P<0.01). However, there were no 2-cell stage embryos obtained from the oocytes with P<1 µm, indicating that a narrow oocyte perivitelline space does not support embryo development (Table 5). Consistent with the activation potential data, oocytes with 1 µm≤P<5 µm showed higher embryo development rates at both the 4-cell and morula stages compared to oocytes with P≥5 µm (4-cell: 32.73% [18/55] versus 7.81% [5/64], P<0.01; morula: 16.36% [9/55] versus 4.69% [3/64]) (P<0.05). However, these two categories of oocytes did not show a statistical difference in the rate of blastocyst formation (7.27% [4/55] versus 3.13% [2/64]) (Table 5). In contrast to the oocyte maturation data, these data suggest that there is an optimal, intermediate perivitelline space width for activation and embryo development.

Our data showed that oocytes with P<1 µm failed to support oocyte maturation, activation, and embryo development. To determine whether this lack of developmental potential is due to the narrow perivitelline space itself or a defect in follicular development, we manually enlarged the perivitelline space of P<1 µm oocytes by removing some ooplasm from the ooctyes with a pipet (Figure 3, bottom line). Although the perivitelline space was enlarged from less than 1 µm to 5 µm following manipulation, the oocyte maturation rate did not improve (1.96% [1/51] versus 2.08% [1/48]). In addition, all of the oocytes, before or after perivitelline enlargement, were unable to be activated (Table 6).

**Table 6:** Effect of enlarging perivitelline space on oocyte maturation and early embryo development.

width ofperivitellinespace (P)	maturation	Repeattimes	PB1%(oocytes withPB1/TotalGV oocytesfor maturation)	2-cell%(2-cells/maturedoocytes foractivation)	4-cell%(4-cells/2-cells)	morula%(morula/2-cells)	blastocyst%(blastocysts/2-cells)
P<1 µm	*In vitro* DO	4	2.08 (1/48)^a^	0 (0/1)^a^	N/A	N/A	N/A
Retrieve partial ooplasm to make	*In vitro* DO	4	1.96 (1/51)^a^	0 (0/1)^a^	N/A	N/A	N/A
1 µm≤P<5 µm							

Within a column, ratio without a common letter differs (P<0.05).

## Discussion

Techniques of human ART, including oocyte IVM, IVF, and ICSI, require high quality oocytes to support development of the fertilized embryo. To obtain multiple mature oocytes for ART, patients are administered gonadotropin. However, some patients do not respond well to gonadotropin stimulation, and other patients suffer strong side-effects. Alternatively, GV stage oocytes can be retrieved from the patient’s ovary and subsequent *in* *vitro* maturation and fertilization can be conducted. There have been several studies focused on the evaluation of matured oocytes [30], [31]. However, for oocytes in the GV stage, practical criteria need to be established to predict their quality and potential for maturation, fertilization, and embryo development. Unlike molecular analysis to analyze specific markers in oocytes, which often requires oocyte destruction, morphological analysis of live GV stage oocytes is more convenient and practical in the clinic. Therefore, we categorized GV stage oocytes according to their morphological traits, including the thicknesses of their cumulus layer and ZP, and the width of their perivitelline space. We found that the developmental potentials of the oocytes varied across the range of each category.

Our results showed that the maturation and developmental potentials of GV stage oocytes were correlated with the thickness of the attached cumulus layer. Oocytes with more cumulus cell layers matured better in culture than those with less cumulus cell layers. These results indicate that the cumulus layer could be used as a morphological marker to predict the quality of GV stage oocytes. The function of cumulus cells surrounding the oocyte is exhibited in three stages: follicle growth, oocyte maturation, and fertilization. As one of several important components in the growing follicle, cumulus cells affect the acquisition of oocyte developmental competency [32]. During follicle development, communication between the cumulus cells and the oocyte provides a mechanism for regulating oocyte growth [33]. This function of cumulus cells may influence not only the nuclear maturation of oocytes, but also the cytoplasmic maturation [34]. Our results showed that oocytes that had fewer cumulus cell layers had a lower potential to undergo maturation, fertilization, and development to an early embryo stage.

Our data also showed that manually removing cumulus cells from the oocytes punctured from matured follicles did not alter the rate of nuclear maturation, or PB1 extrusion. This indicates the oocyte nuclear maturation potential is largely obtained during follicle growth. Increased cumulus cell layers in follicles enable oocytes with the capacity of nuclear maturation, and this inherited capacity is not affected by the removal of cumulus cells from oocytes after they are released from follicles [35]. However, during oocyte *in* *vitro* maturation, cumulus cells continue to promote oocyte cytoplasmic maturation, which is reflected in the embryo development potential of oocytes [36]. This could be the reason that, in our experiments, partial removal of cumulus cell layers during oocyte *in* *vitro* maturation impaired subsequent embryo development.

The cumulus cell layer surrounding oocytes is also important during the process of fertilization. A previous report showed that cumulus cells are essential for sperm-oocyte binding. The junction between cumulus cells and the oocyte also plays an important role in preventing poly-sperm penetration into the oocyte [37]. Another role of the cumulus layer is to promote sperm capacitation and sperm-oocyte binding, as shown by several studies that found that anti-bacterial factors, and other secreted factors, from cumulus cells enhance sperm capacitation and sperm-oocyte recognition [38]–[40]. Thus, multiple functions of cumulus cells make them indispensable during fertilization. Other suggested functions include the mutual recognition between the oocyte and sperm [41], guiding the sperm to the oocyte, inducing sperm capacitation and the acrosome reaction, maintaining sperm motility, dispersing sperm to prevent polyspermy, preventing ZP hardening, and improving sperm penetration into the oocyte. Consistent with these data, our results showed that artificial removal of cumulus cell layers from oocytes resulted in significantly reduced fertilization rates, further indicating that the cumulus layer is essential for the fertilization process.

In our study, ZP thickness was used as another morphological criterion to evaluate GV stage oocyte quality. Our results showed that oocytes with an intermediate ZP thickness had the highest maturation rate. The nuclear maturation rate was decreased if ZP thickness was more than 8 µm, but this increased ZP thickness did not affect the potential of fertilization or early embryo development. On the contrary, when oocytes with the thinnest ZP (<5 µm) were cultured, few of them matured, and none of them could be fertilized. Our results indicate the parameter of ZP thickness can be used to evaluate oocyte quality.

The ZP plays an important role during fertilization, early embryo development, and implantation [42]. The ZP first appears in the unilaminar primary follicle and is secreted by both the oocyte and the follicular cells. The thickness of the ZP may reflect the stage of follicle growth. It is possible that oocytes with a thin ZP come from immature follicles, which leads to their failure in maturation and subsequent fertilization. This would be consistent with our results showing that an oocyte with a ZP thickness less than 5 µm fails to undergo maturation. On the other hand, previous research has shown that human age influences ZP thickness. The oocytes from older women have thicker ZPs than those from younger women [43], [44]. Thus, oocytes with thick ZPs may indicate that the oocyte is aged, which could also impair the maturation potential. In our study, partial digestion of the ZP with tyrode solution to decrease its thickness, resulted in an increased oocyte maturation rate. We believe that it is unlikely that the maturation potential of an aged oocyte with a thick ZP could be reversed by a simple “ZP thinning” treatment. However, our results did show that this treatment enhanced the nuclear maturation of oocytes that previously had thick ZPs. Perhaps this is due to some sort of activation of the aged oocyte maturation signals by the acid solution. Alternatively, thinner ZPs could simply facilitate PB1 extrusion. This interesting phenomenon needs to be investigated further.

The ZP is also essential for fertilization. As receptors for sperm recognition, the ZP glycoproteins, including ZP1, ZP2, and ZP3, can be combined with sperm to trigger the acrosome reaction and complete subsequent fertilization [45], [46]. A recent study showed that ZP lacking functional ZP1 cannot be fertilized [21]. Others have shown that abnormalities in the ZP lead to a failure in fertilization [47]. In our study, because ZP thickness can only be visualized and measured in denuded oocytes without the attached cumulus cells, it was necessary to use chemical-induced activation for 2-cell and later embryo development evaluation of these oocytes. Our data showed that oocytes with a ZP thickness greater than 5 µm had a higher potential to be activated and to develop to the blastocyst stage, and that this potential could not be improved or impaired by manually changing ZP thickness. These results illustrate that ZP thickness can be used to evaluate oocyte maturation in the follicle, and to determine the potential for activation and embryo development of oocytes.

In our study, the third criterion used for evaluating GV stage oocyte quality was the width of the perivitelline space. Our results showed that wide perivitelline spaces enhanced oocyte PB1 extrusion. Conversely, wide perivitelline spaces impaired the rates of 2-cell, 4-cell, and morula formation. On the other hand, oocytes with a very narrow perivitelline space (P<1 µm) had a very low maturation rate, and failed to be activated, and these effects could not be reversed by manual enlargement of the perivitelline space.

So far, two methods for perivitelline space enlargement in oocyte were reported. One is increasing the osmotic pressure of the culture medium, the other is removing partial ooplasm from oocyte. Previous report from other’s study showed that changing osmotic pressure is deleterious to the oocyte maturation [48]. Thus, this protocol was not employed in our study. For the second method, in our previous published data, protocol of retrieving partial ooplasm from oocytes was used to investigate effects of ooplasm transfer on paternal genome function in mice. Data from this report illustrate early embryo development is not affected by this manipulation [49]. However, our subsequent study demonstrate ooplasm transfer itself was associated with reduced viability in live pups [50]. Thus, we believe extracting partial cytoplasm from oocyte will affect oocyte quality. And this may exert its effect on later development of offspring. In the current study, early stage embryo development was investigated for the ratio calculation.

The width of the perivitelline space changes during oocyte growth, fertilization, and early embryonic development [51]. The perivitelline space contains hyaluronic acid and extracellular matrix prior to fertilization. During oocyte maturation, the cortical granules are limited to the oocyte cortex and the electron density of the ZP increases [52]. As oocytes mature and move to the fallopian tube, a substance released from the cortical granules plays role in the inner half layer of the ZP, resulting in an increase in the electron density of the ZP [53]. During fertilization, the cortical reaction releases substances into the perivitelline space. These substances play a key role in fertilization, as indicated by a previous report showing that oocytes lacking cortical granules cannot be fertilized [54]. The cortical granule envelope exists until blastocyst hatching, implying that it also has an important role in blastocyst hatching [51]. In our study, oocytes with a narrow perivitelline space (P<1 µm), showed poor maturation potential and failed to undergo activation. We believe that the narrow perivitelline space could be indicative of insufficient cortical granules, which would subsequently lead to poor oocyte quality. Conversely, our data showed that oocytes with a larger perivitelline space width (P≥5 µm) had a higher potential for PB1 extrusion, potentially because the larger perivitelline space provides more room for PB1 extrusion. In contrast, the oocytes with a larger perivitelline space width had lower activation and embryo development potentials relative to ooytes with an intermediate perivitelline space width. Perhaps, when the perivitelline space is too large, the concentration of released cortical granule substances and extracellular matrix is below that which is required for proper oocyte development. Another reason could be that a GV stage oocyte with a larger perivitelline space has less cytoplasm, and thus fewer nutrients to support subsequent embryo development.

Artificial enlargement of perivitelline space width by extracting a portion of the cytoplasm from immature GV stage oocytes failed to improve maturation and embryo developmental potentials. We speculate that the width of the perivitelline space reflects the degree of oocyte maturity. Thus, the cortical granules or degree of oocyte maturity cannot be changed by mechanically enlarging the perivitelline space.

In conclusion, our study provides detailed morphological methods for selecting GV stage oocytes with high developmental potential. Oocyte quality in the GV stage has a strong influence on maturation, fertilization, and later embryonic development. A convenient assessment method, including measuring the thicknesses of the cumulus layer, ZP, and perivitelline space, will provide an additional opportunity to improve human assisted reproductive techniques.

## Supporting Information

Checklist S1 **ARRIVE Guidelines Checklist.**
(DOC)
